# Bacteriophage Tail Proteins as a Tool for Bacterial Pathogen Recognition—A Literature Review

**DOI:** 10.3390/antibiotics11050555

**Published:** 2022-04-21

**Authors:** Karolina Filik, Bożena Szermer-Olearnik, Sabina Oleksy, Jan Brykała, Ewa Brzozowska

**Affiliations:** Hirszfeld Institute of Immunology and Experimental Therapy, Polish Academy of Sciences, St. R. Weigl 12, 51-167 Wroclaw, Poland; oleksy.sabina01@gmail.com (S.O.); janbrykala@gmail.com (J.B.); ewa.brzozowska@hirszfeld.pl (E.B.)

**Keywords:** RBP, tail fiber protein, pathogens detection, bacteriophages, diagnostic

## Abstract

In recent years, a number of bacterial detection methods have been developed to replace time-consuming culture methods. One interesting approach is to mobilize the ability of phage tail proteins to recognize and bind to bacterial hosts. In this paper, the authors provide an overview of the current methodologies in which phage proteins play major roles in detecting pathogenic bacteria. Authors focus on proteins capable of recognizing highly pathogenic strains, such as *Acinetobacter baumannii*, *Campylobacter* spp., *Yersinia pestis*, *Pseudomonas aeruginosa*, *Listeria monocytogenes*, *Staphylococcus aureus*, *Enterococcus* spp., *Salmonella* spp., and *Shigella*. These pathogens may be diagnosed by capture-based detection methods involving the use of phage protein-coated nanoparticles, ELISA (enzyme-linked immunosorbent assay)-based methods, or biosensors. The reviewed studies show that phage proteins are becoming an important diagnostic tool due to the discovery of new phages and the increasing knowledge of understanding the specificity and functions of phage tail proteins.

## 1. Introduction

Bacteriophages represent the most abundant life form on Earth and can be found in all environments in which bacteria grow. Phages are detected in ground and surface water, soil, food, sewage, as a component of human and animal microbiomes, etc. [1]. They are responsible for 10–80% of the total bacterial mortality in aquatic ecosystems and are an important factor limiting bacterial populations [2,3]. A characteristic feature of bacteriophages is their affinity for specific bacteria, which arises through their ability to specifically recognize molecules present on the host bacterial surface. This property has been successfully used for bacterial diagnosis and identification [4]. The interactions between phages and their hosts depend on the phage morphology, bacterial surface structures, and environment. The so-called monovalent bacteriophages infect a specific strain of bacteria, while the polyvalent bacteriophages show specificity towards several bacterial strains [5]. The specific molecules through which bacteriophages bind to the bacterial cell surface include lipopolysaccharide (LPS), fimbriae, flagella, and various proteins [6]. Bacteriophages that infect encapsulated bacteria need to break down the capsule barrier to reach their cell-surface receptor. These phages bind to polysaccharides of the capsule and mimic an enzyme-substrate reaction to degrade the cell envelope. Some bacteriophage-generated enzymes diffuse into the medium, strip the cell sheaths from around the plaques, and induce a “halo” zone [7]. The degrading enzymes may be part of the phage tail, such as the tail tubular proteins (TTP) [8,9]. The varied structural composition of the bacterial cell surface means that there is a wide range of molecules that can act as receptors. On Gram-positive bacteria, potential recognition roles are played by cell wall elements such as teichoic acids and lipoteichoic acids; on Gram-negative bacteria, the targeting molecules include LPS, outer membrane proteins (e.g., porins), pili, and flagella [6,10]. Kinetically, the adsorption process is a first-order reaction whose rate is directly proportional to the concentrations of both phage and bacteria. In an environment where phages dominate, even several hundred phage particles can adsorb on a single host cell [11]. The adsorption constant can be calculated theoretically as a function of the virus diffusion rate (as a factor leading to virus-cell collision), virus dimensions, environmental viscosity, and environmental temperature [12].

Prior to phage adsorption, the bacterial cell surface must be modified through the involvement of cations and (in some cases) the attachment of tryptophan or another cofactor, such as phenylalanine, tyrosine, or diiodotyrosine. Some agents, such as 2-pyridylalanine and 3-pyridylalanine, facilitate the adsorption of the phage to the bacteria [13]. The presence of electrolytes in the culture medium both supports the adsorption of phage particles to bacteria and influences the effective infection of host cell [13,14]. The presence of calcium, magnesium, and barium ions may inhibit the activity of some phages, while others seem to require calcium ions for virus adsorption [15]. In the absence of electrolytes, adsorption can be reversible and non-infective. Low-molecular-weight organic compounds can attach to and activate phage particles. For example, six tryptophan molecules are necessary to activate bacteriophage T4 [16]. Indole, on the other hand, inhibits the adsorption of a bacteriophage to the bacterial cell. Organic compounds may alter the conformation of sites responsible for the bacterial cell binding of a phage, thereby facilitating or preventing adsorption [17].

**Figure 1:**
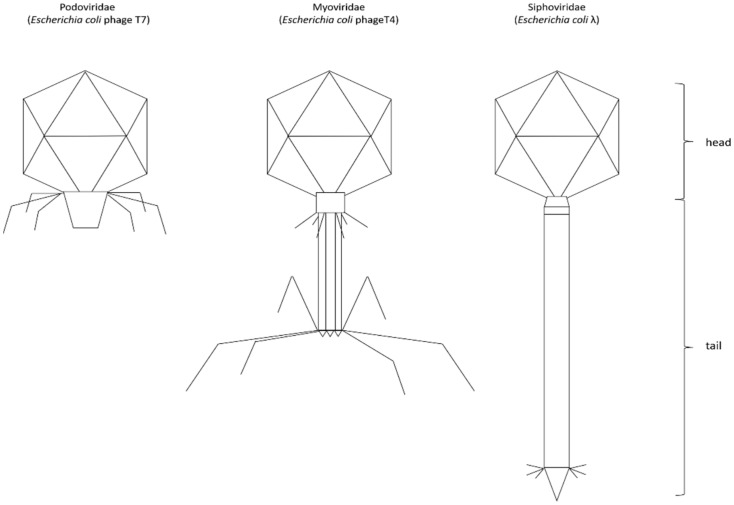
Classification of the *Caudovirales* based on the tail structure.

Tailed phages of order *Caudovirales* can be classified into three groups on the basis of their tail structures (Figure 1), which relate to the structures of their target capsids. Phages having a short, non-contractile tail are classified as *Podoviridae*; phages with a contractile tail are classified as *Myoviridae*; and those with a long but non-contractile tail are classified as *Siphoviridae* [10,18].

Phage tails show high specificity and fulfill key tasks to enable phage infection. The phage tail and its RBP (receptor binding proteins) critically govern host recognition via the specific interaction between attachment sites of the tail and molecules on the bacterial surface. To recognize receptors on the surface of bacterial cells, tailed phages use either a set of tail fibers (TF; there may be 3, 6 or 12 fibers) or a single TF located in the center of the baseplate. The adsorption of RBPs to specific bacterial surface molecules is reversible at the beginning and turns into irreversible interaction [10]. The model phage of *Podoviridae* is *Escherichia coli* phage T7. One vertex of its icosahedron capsid protein, gp8, forms a portal that contributes to DNA transport and also acts as a connector for the tail. The tail comprises six molecules of gp12 and 12–18 copies of gp11. The tail fibers are localized on the tail just below where it joins with the capsid. The phage has six tail fibers, each of which comprises a gp17 trimer [19]. One of the most extensively studied model phages is the T4 bacteriophage, which belongs to the *Myoviridae* family. The T4 tail has the most complex morphology among the Caudovirales. Structurally, the tail is attached to the head by the collar, which sprouts six whiskers. These whiskers are encoded by the gene *wac* (whisker antigen control); they comprise fibrin protein, which plays a crucial role in correct tail assembly during the final phase of phage infection. Fibrin moieties attach to the head of the fiberless phage and form the collar. The whiskers interact with the LTF (long tail fibers), stimulating the assembly and attachment of the LTF to the baseplate [20]. The next part of the tail is the tube, which consists of an internal tube (gp19) and a sheath that is built around the internal tube from gp18 subunits [21,22]. At the distal part of the tube is a baseplate with attached LTF [22,23]. The baseplate consists of gp9, gp10, gp11, and gp12. The long tail fiber consists of gp34, gp35, gp36, and gp37. In T4 phage, the long tail fibers take part in host recognition through a reversible interaction of their tips with LPS or porin. The whiskers (also called short tail fibers) are responsible for an irreversible interaction: upon receptor binding, a recognition signal sent to the baseplate causes the whiskers to extend and bind to the core region of LPS [24]. The model phage of *Siphoviridae* is *E. coli* phage λ, which has a long tail that consists of a tube made from gpV. The major role of the tube is DNA transport and injection during infection. At the distal part of the tube, there is a single tail fiber composed of gpJ. The fiber facilitates the attachment of phage λ to the host cell by binding to LamB on the bacterial envelope surface [25].

Once successful binding to the host receptor has occurred, a conformational alteration in the phage’s baseplate takes place, leading to sheath contraction and injection of the phage’s nucleic acids into the host cell [6]. The RBPs include phage tailspike, tail fiber, and spike proteins. Due to their diverse host-binding specificities, RBP-encoding genes are often missed by genomic sequence analyses based solely on homology with known RBPs. Even if phage RBPs share structural homology, they tend to lack sequence homology. RBPs have been shown to be very stable proteins with high resistance to proteases and detergents [26]. Overall, the high stability, specificity, and ease of recombinant overexpression make RBPs excellent alternatives to antibodies and ideal tools for the development of new diagnostic technologies.

## 2. Overview of Methodologies That Use Bacteriophage Tail Proteins for Detecting Pathogenic Bacteria

**Figure 2:**
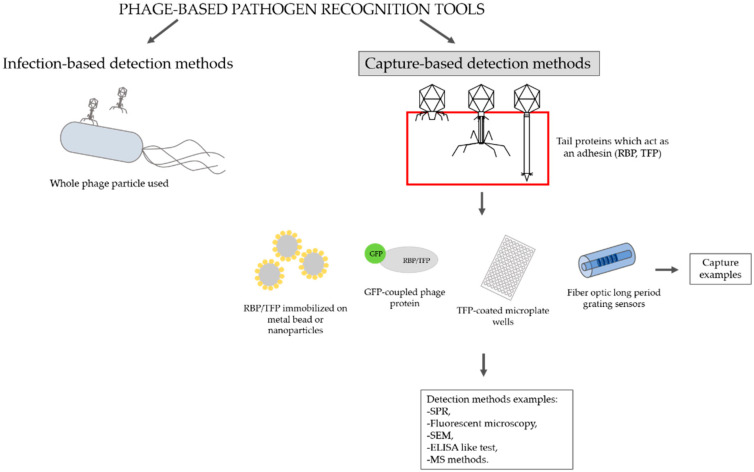
Phage-based pathogen recognition tools. SPR—surface plasmon resonance; SEM—scanning electron microscopy; ELISA—enzyme-linked immunosorbent assay; MS—mass spectrometry.

For diagnostic applications, whole phages and phage-derived proteins have been extensively explored [27]. According to Meile et al. (2020), phage-based pathogen recognition tools can be divided into two categories by their modes of action: infection-based and capture-based detection methods (Figure 2). In the present review, we focus on capture-based detection methods in which tail proteins are used as bio-sensing molecules to detect a particular bacterial strain. In the following, we describe the relevant methodologies that may be implemented for bacterial detection.

Whole phage particles can be applied as bio-recognition elements through their specific binding to the host. This approach has been extensively explored in the development of biosensors. A biosensor can be defined as a detection device in which biomolecular interactions between a bioprobe and an analyte are translated into a measurable signal by means of transduction systems [28]. A typical biosensor consists of a surface functionalized with a biorecognition element, a transduction system that generates a signal reflecting the number of binding events, and an amplifier that processes the signal to give a readable output [29]. The bioprobe is arguably the most crucial element: it confers specificity and sensitivity, which are essential features of a reliable diagnostic tool. Various types of molecules have been used for this purpose, including nucleic acids [30], antibodies or antibody fragments [31,32], and both wild-type and engineered phages (reporter phages) [33]. Another group of potential bioprobes comprises phage-derived binders, such as RBP (described in more detail below) [34,35], endolysin cell wall-binding domains (CBD) [36,37,38], and phage-display peptides [39,40]. Details may be found in existing comprehensive reviews of biosensor transduction systems and their modes of action, e.g., [41].

In addition to surface-immobilized phages in biosensors, whole phage particles, conjugated with, e.g., magnetic nanobeads can also be used as capture probes in bacterial detection assays. However, the large size and possible lytic properties of whole phages may complicate such efforts [42]. Accordingly, researchers have sought to apply phage-derived affinity molecules, such as tail fiber or tailspike RBP and some endolysin CBD. As there are considerable differences in the cell envelopes of Gram-negative and Gram-positive bacteria, it is no wonder that the cell wall-targeting endolysins evolved different structures: phages that infect Gram-positive hosts possess endolysins with both an enzymatically active domain (EAD) and a CBD, whereas those that infect Gram-negative bacteria have endolysins in the dominant majority consisting of a single EAD [43]. CBD are believed to specifically recognize peptidoglycan modifications and decorations, which are characteristic of and common in Gram-positive bacteria [44,45,46,47]. However, due to the conserved nature of peptidoglycan, the mechanisms of enzymatic cleavage are much more limited; only five classes of endolysins have been described to date [48]. The above has several implications for the application of endolysin for bacterial detection: (1) only endolysins from phages of Gram-positive hosts can be readily used for bacterial detection and, conversely, only Gram-positive bacteria can be detected with this approach; (2) the separation of the recognition element and enzymatic activity facilitates the development of a single domain into a bioprobe; and (3) the high specificity of recognition by CBD yields a desirably narrow host range. The feasibility of CBD-based pathogen recognition has been reported by multiple research groups. For instance, endolysin CBD fused with fluorescent proteins have been applied as labeling probes in fluorescence microscopy [49,50,51,52]. CBD has been coated onto magnetic beads for the separation and enrichment of bacteria, followed by culture-based enumeration [53,54], polymerase chain reaction (PCR)-based detection [53], the use of reporter phage [55], commercial ATP bioluminescence assays [56], or staining with fluorescently labeled CBD [49]. The development of a lateral flow assay (strip test) employing fusion proteins of an endolysin CBD to detect *Bacillus cereus* has also been reported [57]. Table 1 summarizes the methodologies in which phage tail proteins have been used to detect host bacteria.

### 2.1. Acinetobacter baumannii Detection

*Acinetobacter baumannii*, considered to be one of the opportunistic pathogens, is notorious for its propensity to cause nosocomial infections, including sepsis and ICU (Intensive Care Unit)-acquired pneumonia [71]. It is one of the ESKAPE pathogens, which are a group of highly virulent bacteria with high antimicrobial resistance that are able to “escape” the harmful effects of antibiotics and cause serious infections [72,73]. The success of this pathogen has further been attributed to its outstanding ability to withstand harsh environmental conditions. This is achieved in part through surface glycans such as CPS (capsular polysaccharide), which is composed of multiple repeating oligosaccharide units [74]. The CPS structure called the K type has been elucidated for multiple *A. baumannii* isolates, but no comprehensive serotype classification has been developed to date [75]. The extraordinary diversity of the *A. baumannii* CPS is reflected by the existence of at least 128 different gene clusters (KL clusters) responsible for CPS synthesis and export [76]. Importantly, CPS is targeted by phage RBP during adsorption [77]. Increasing evidence suggests that the K type is an important specificity determinant, with many *A. baumannii* phages infecting strains of a single or very limited number of K types [78,79,80,81,82].

Many groups have studied *A. baumannii* phage tail fiber/tailspike proteins with a particular focus on their depolymerase activity and potential as a prospective antivirus treatment [83,84,85,86,87,88]. Below, we outline research attempts to harness the specificity of RBPs for detecting *A. baumannii.* The extensive diversity of CPSs in this pathogen limits the feasibility of a method aimed at its rapid and broad-range detection. A more feasible application for phage RBPs in this case might be using them to develop a typing scheme. Xu et al. (2020) recently described the isolation of a new phage capable of infecting five strains of *A. baumannii*. The authors identified two putative tail fiber proteins (TFPs) in genomes of these phages and demonstrated that they bind susceptible cells and lack lytic activity, and thus could be useful as detection tools. To examine the target specificity of the TFPs, the authors devised a sandwich fluorescent assay. Microplate wells were coated with non-labeled TFPs to capture bacterial cells, and bacteria were subsequently detected using analogous, FITC (fluorescein isothiocyanate)-labeled proteins and fluorescence microscopy. The probes exhibited good specificity, as they did not recognize selected strains of *E. coli*, *Acinetobacter haemolyticus* or *Bacillus subtilis*, but they did recognize a susceptible *A. baumannii* strain in a mixed culture. For a detection assay, bacteria were incubated with TFPs conjugated to magnetic beads and the results were visualized using a bioluminescence assay with a commercial luciferin/luciferase kit that measured intracellular ATP release. The authors claimed that an optimized protocol enabled them to detect as few as 6.2 × 10^2^ CFU/mL (colony-forming unit per mililiter) *A. baumannii*. They investigated the clinical applicability of this technique by testing artificially contaminated human samples (urine, sputum, feces), and found that 50–93% of the target bacteria could be recovered from the sample, depending on the sample type and bacterial load [58].

Bai et al. (2019) reported an approach in which alumina-coated magnetic nanoparticles were used to immobilize two distinct TFPs (TF2 and TF6) that selectively recognized two clinical strains of *A. baumannii*. Binding efficiency was evaluated in terms of the change in optical density (OD600) between readouts taken before and after incubation with a nanoparticle probe. The OD change was significantly higher for target bacteria than for non-target bacteria. TEM (transmission electron microscopy) observations showed significant aggregation of nanoparticles, but their binding to bacteria was also apparent.

For the detection of *A. baumannii*, the authors combined nanoparticle trapping with MALDI-TOF MS (matrix-assisted laser desorption-ionization time-of-flight mass spectrometry) analysis to uncover a protein fingerprint for further identification. With this approach, the two *A. baumannii* strains were distinguished from one another as well as from other pathogens (*E. coli* O157:H7 and *Staphylococcus aureus*) in a mixed sample. Additionally, the TFP-coated nanoparticles allowed the sample to be enriched to a concentration of bacteria detectable by MALDI-TOF MS. The level of detection for the method was 2.34 × 10^5^ or 4.48 × 10^4^ CFU/mL, depending on the strain. The method was able to detect low concentrations of bacteria (5 × 10^5^–2 × 10^6^ CFU/ml) in diluted fetal bovine serum, supporting its clinical potential. The authors did not examine lower bacterial concentrations, presumably because high ion peaks resulting from the decaying probes would easily obscure the peaks from bacteria present in a sample at a lower concentration. However, MALDI alone, without the selective enrichment, did not detect bacteria present at such a concentration.

To further demonstrate the selectivity of TF2-coated nanoparticles, the authors examined 10 clinical strains: four strains that had been previously described to bind TF2 and six non-binding strains. Bound cells were recovered from the nanoparticles and the CFU of each strain per mg of protein was compared to the value for the original target strain. At a cut-off value established by the authors (≥70% recovery indicating a positive result, <70% indicating a negative result), TF2-coated nanoparticles could differentiate between known TF2-binding and non-binding strains. [59].

The *A. baumannii* ΦAB6 phage tailspike protein, TF6 (ΦAB6TSP), has attracted attention due to its ability to recognize and hydrolyze bacterial surface glycans containing pseudaminic acid (Pse) [89]. Pse has been identified in numerous Gram-negative pathogens, including *Campylobacter jejuni* and *Helicobacter pylori*, and it has commonly been implicated in virulence [90]. In *A. baumannii* strains, the sequence encoding Pse was identified in two capsule biosynthesis gene clusters [91]. The use of this particular TSP could, in principle, allow the detection of various Pse-coated bacteria. This was recently investigated by Lee et al. (2020). Because the enzymatic activity of Pse could hinder downstream detection, the authors prepared an inactive mutant protein that retained its target specificity. The corresponding inactivated fluorophore-conjugated TSP exhibited a strong relationship between signal intensity and probe/bacterial concentration. The TSP-based probe was deemed to be more sensitive in detecting a susceptible *A. baumannii* strain than an antibody raised against EPS (exopolysaccharide) hydrolysis products containing Pse. The authors also attempted the detection of other Pse-coated bacteria (two strains of *H. pylori* and one of *Enterobacter cloacae*). These strains showed a similar binding capacity, as reflected by fluorescence microscopy observations and the identification of a linear relationship between the fluorescence intensity and optical density of bacterial suspensions. The authors further developed an assay for detecting Pse-coated bacteria by immobilizing the TSP onto the wells of a microplate and measuring the fluorescence of FITC-labeled, bound bacteria. They observed a linear relationship between the amount of bacteria and the fluorescence intensity for Pse-containing strains, but not for Pse-lacking strains. However, the authors noted that the labeling and immobilization steps can introduce significant variation in the results, limiting the usefulness of this technique [60].

### 2.2. Campylobacter *spp.* Detection

*Campylobacter* spp. is a Gram-negative foodborne bacterial pathogen. The first report of *Campylobacter* spp. dates back to 1886, when Theodore Escherich observed non-culturable spiral-shaped bacteria. The hosts of this pathogen include both wild and domestic animals; it is frequent among birds, especially poultry, probably because of their higher body temperature [92]. Chicken products have been implicated in a large number of *Campylobacter* spp. infections in human populations, due to the high consumption of chicken meat [93,94,95,96]. *Campylobacter* spp. is also commonly found in other livestock, such as cattle, pigs, cows, lambs, ducks, and turkeys [97]. Consumption of untreated water is also considered a risk factor [93,98]. *Campylobacter* spp. causes mild to serious infections of children and the elderly, called campylobacteriosis. The most common symptom is diarrhea, but the infection may lead to permanent neurological damage, such as that of Guillain–Barré syndrome [93]. For *Campylobacter* and certain other species, viable but non-culturable cells (VBNC) can exist (but not replicate) under unfavorable growth conditions. Cells in this state can still infect susceptible hosts [93,99].

Poshtiban et al. (2013) proposed a platform that used the bacteriophage tailspike protein, GST-Gp48, to detect pathogenic *C. jejuni* via an interesting biosensor-based method [64,100]. They employed a micromechanical resonator that enabled the high-throughput and label-free diagnostic analysis of multiple samples. During the experiment, the device monitors the resonance frequency shift, which depends on the mass of the adsorbed target analyte. The microresonator was functionalized with the GST-Gp48 tailspike protein, and the specific capture and detection of *C. jejuni* cells was demonstrated. This microresonator array was highly mass sensitive and had a large surface-to-volume ratio. The detection and quantification of *C. jejuni* cells were confirmed by an SEM (Scanning Electron Microscope). The simulations of resonance behaviors were tested using Finite Element Analysis (FEA). The results of these simulations showed that the frequency shift was determined by the mass of bacteria captured on the microresonator surface. The authors used SEM visualization to calculate the number of bacteria attached to the sensor and found that 225 ± 13 bacteria bound to a single element on average. The specificity of capture was established by comparing binding between *C. jejuni* and *E. coli* cells (negative control). The findings were promising, and the method seemed to be relatively inexpensive and rapid compared to conventional methods. This microresonator-based biosensor enabled the highly specific detection of bacteria in a sample with a low bacterial load. Moreover, microresonator arrays do not require sample pre-enrichment and the experiments are label-free [64].

Two methods for detecting *C. jejuni* and *Campylobacter coli* were reported by Javed et al. (2013). The authors described a phage RBP-based agglutination assay and a method of GFP-coupled RBP probe detection combined with fluorescence microscopy. First, the team identified an RBP (Gp047) from *C. jejuni* phage NCTC12673 and established that its C-terminal domain was responsible for its specific host recognition. They found that *C. jejuni* NCTC11168 cells mixed with CC-Gp047 formed aggregates within 1 min on a glass slide. The authors confirmed that the observed aggregates were caused by probe binding and not by autoagglutination of bacterial cells. The agglutination test was performed on a wide range of bacterial species (*C. jejuni*, *C. coli*, *Campylobacter lari*, *Campylobacter fetus*, *Campylobacter fetus venerealis*, *Campylobacter concisus*, *Campylobacter upsaliensis*, *H. pylori*, *E. coli*, and *Salmonella enterica*). The results indicated that agglutination also occurred efficiently when the cells were in the VBNC state. To check the robustness of the method under different conditions, different growth media (MH, BHI, LB, and NCZYM) and buffers (phosphate buffer pH 7.4, HEPES buffer pH 7.4, standard saline, Tris-HCl buffer, pH 7.5, and 5% BSA in PBS) were examined. The agglutination results were the same for all tested conditions. The RBP-based agglutination assay showed 100% specificity for both *Campylobacter* species, and 95% and 90% sensitivity for *C. jejuni* and *C. coli*, respectively. Importantly, agglutination tests are rapid and do not require sophisticated equipment (only a simple glass slide) [65].

For the second method, the authors used GFP-fused CC-Gp047 and fluorescence microscopy for detection. Bacterial suspensions (*C. jejuni* NCTC11168, *C. coli* RM2228, and *E. coli* DH5α) were incubated with the labeled protein and microscopic observations were carried out. *C. jejuni* and *C. coli* showed green fluorescence, indicating the binding of EGFP_CC-Gp047, while *E. coli* DH5a did not. As a follow-up experiment, the authors tested a mixed culture of bacteria containing *C. jejuni* NCTC11168 and *E. coli* DH5a at a cell number ratio of approximately 1:25. Only *C. jejuni* cells (as discerned by characteristic morphology) showed green fluorescence under microscopic observation. The RBP-based probe, EGFP_CC-Gp047, combined with fluorescence microscopy was thus able to detect low numbers of *C. jejuni* and *C. coli* cells in a mixed culture. Both of the introduced methods are simple and specific and can be used for the simultaneous detection of pathogenic *Campylobacter* species [65].

### 2.3. Listeria monocytogenes Detection

*Listeria monocytogenes* is a major public health concern in the food industry due to its ability to survive the harsh conditions commonly applied in food processing and preservation, such as low temperature, high salt concentration, dehydration, and extreme pH [101]. Infection with *L. monocytogenes*, known as listeriosis, occurs as a result of ingesting contaminated foods; reported outbreaks of listeriosis have been mainly connected to dairy products, meat, fish, fruits, and vegetables [102]. Listeriosis generally presents as gastroenteritis, but in the case of vulnerable individuals (including children, the elderly, pregnant women, and immunocompromised individuals), it poses a threat of sepsis, meningitis, and premature termination of pregnancy [103]. Given the severity of symptoms in the susceptible population and the finding of a lower infectious dose than previously suspected, the U.S. Food and Drug Administration (FDA) adopted a “zero-tolerance policy” for this pathogen in ready-to-eat foods [104]. In contrast, the current EU (European Union) regulations accept a tolerable limit of 100 CFU/g for foods that do not support the growth of *L. monocytogenes* as they enter the market, as well as those that do support the growth of this pathogen over the product’s shelf life [105].

Currently, the detection of *L. monocytogenes* and other *Listeria* spp. in food samples is commonly based on culture methods [101]. As a general rule, one or two enrichment steps (i.e., incubation in a selective liquid medium) are required to enrich the target bacteria to a detectable concentration and inhibit the growth of competing microflora. Cultures are then plated onto selective differential or chromogenic media and the resulting colonies are examined for characteristic morphology. As a slow-growing microorganism, *Listeria* spp. requires extended incubation times, making this a relatively slow process. Thus, new, rapid, and robust detection methods are needed. Numerous nucleic acid-based techniques have been applied to the detection of *L. monocytogenes*, including PCR, multiplex PCR, real time/quantitative PCR (qPCR), loop-mediated isothermal amplification (LAMP), and whole-genome sequencing analyses. For a more detailed description of representative culture methods and novel molecular approaches, the interested reader is referred to the review by Law et al. (2015). These molecular techniques are extremely sensitive and specific; however, they are generally unable to discriminate between viable and inactivated microorganisms, resulting in false positives [106]. Meanwhile, the harsh conditions of food processing are expected to stress bacterial cells, causing them to become non-cultivable on selective media and leading to their underestimation in culture-based methods [107].

*L. monocytogenes* is currently classified into 12 serotypes; of them, serotypes 1/2a, 1/2b, and 4b are the most commonly associated with clinical disease [108]. The serotypes are determined primarily by the structure of the cell wall-associated carbohydrate polymer, teichoic acid, which is specifically recognized during phage adsorption by RBP [109]. Phages of Gram-positive hosts, including *L. monocytogenes*, possess endolysins (peptidoglycan-cleaving enzymes) with CBDs that exhibit remarkable specificity towards peptidoglycan-associated molecules, including teichoic acid [48,110]. The selectiveness of both types of phage-derived proteins supports their possible application in diagnostic and/or serotyping tools.

The phage-based methods reported to date for detecting *L. monocytogenes* have focused primarily on endolysins [38,49,53,54] and broad-range *Listeria* reporter phages [55,111,112]. A commercially available rapid and semi-automated phage protein-based test for detecting *Listeria* spp. was developed and validated (VIDAS UP Listeria, bioMérieux) [112]. Based on the principle of enzyme-linked fluorescent assay, this method uses alkaline phosphatase-conjugated protein probes to capture target bacteria onto a solid surface and direct substrate cleavage to enable fluorescence detection [113]. However, the exact identity of the utilized phage proteins has not been disclosed.

The applicability of phage TFP and endolysins for the diagnosis and typing of *L. monocytogenes* was recently demonstrated by Sumrall et al. (2020). The authors gathered a set of six proteins (three RBP and three endolysin-derived CBD) from a collection of *Listeria* phages with the aim of developing a quick, reliable, and objective serotyping method. In this scheme, GFP-tagged recombinant proteins were incubated with bacteria on a microplate and the fluorescence from bound probes was measured to provide a serovar-specific fingerprint in ~1 h. To verify the accuracy of this novel glycotyping scheme, the authors tested 60 strains of known serotypes; of them, 58 were correctly assigned by the method. Unlike traditional serotyping with a slide agglutination test, which relies on the polyvalent binding of antibodies in serum, this technique relies on precisely described interactions with specific sugar moieties. Importantly, it eliminated the potential discrepancies that could be caused by the subjective interpretation of results or the use of serum preparations from different batches. It also circumvented some of the limitations of PCR-based typing, as it differentiated between live and inactivated bacteria as well as between closely related serotypes arising from a single point mutation.

To further illustrate the usefulness of the phage protein-based detection and differentiation of *L. monocytogenes*, the authors developed a method for differentially separating the most common pathogenic serotypes: 1/2 and 4b. Paramagnetic beads were functionalized with one of two selected GFP-tagged RBPs to allow for the selective enrichment and detection of these serotypes under fluorescence microscopy. The probe-bound beads were found to be efficient at separating the target serotypes in a mixed culture, although the binding affinity of the 4b-specific beads was significantly lower than that of the 1/2-specific ones. This finding led the authors to develop avidin-tagged directionally coupled probes that exhibited significantly better affinity but lacked the GFP tag essential for fluorescence-based detection. When tested against a comprehensive library of strains of different serotypes, both probes exhibited good specificity. Surprisingly, the 4b-targeting beads also bound nonpathogenic *Listeria innocua* serotype 6a and *L. monocytogenes* 4e, even though the latter harbored few target glycan molecules. These results suggest that phage tail proteins could be a useful tool for developing a rapid and reliable diagnostic assay for *L. monocytogenes* [109].

A different strategy for detecting *Listeria* spp., *E. coli* O157:H7, and *Salmonella* spp. was implemented by Junillon et al. (2012). The authors developed a simple device comprising a polystyrene surface coated with phage proteins and a colorimetric reaction to visualize bound bacteria. The detection was carried out directly in homogenized food samples over the course of a standard enrichment-period incubation (22–40 h depending on the strain) in a liquid medium. The visualization was based on the reduction of colorless triphenyltetrazolium chloride. The resulting red formazan crystals accumulated inside the cells, giving the sensor surface a colored appearance upon binding. To test the applicability of this method, the authors incubated homogenates with approximately 5 CFU of a given strain in the presence of the detection device. The test gave a strong positive result for *L. monocytogenes* serotype 4b and *Listeria seeligeri* in roast pork. Of note, this test used a broad-range phage protein and thus did not discern between different *Listeria* species [114].

### 2.4. Yersinia pestis Detection

*Yersinia pestis* is a highly pathogenic Gram-negative bacterium that is the causative agent for plague. Historically, this serious and potentially deadly zoonotic disease was responsible for pandemics occurring in the mid-6th, mid-14th, and early 20th centuries [63,115]. *Y. pestis*, which is a member of the *Enterobacteriaceae* family, is a nonmotile, non-spore-forming coccobacillus. Its growth temperature ranges from 4 to 40 °C, with an optimum range of 28–30 °C. The high pathogenicity of this bacterium reflects its ability to adapt to temperature changes: it can survive and replicate in both cold-blooded insects (fleas with body temperature of 20–28 °C) and warm-blooded mammals. Moreover, the bacterium can form a gel-like protective capsule with antiphagocytic properties. Humans can become infected after being bitten by a rodent-hosted flea or by direct contact with animals suffering from plague. Three clinical forms of plague can be distinguished: bubonic, pneumonic, and septicemic. Pneumonic plague is the most serious form of the disease, and the only one that can be transmitted from person to person. Due to its highly contagious nature, *Y. pestis* is currently detected with the following methods: ELISA (enzyme-linked immunosorbent assay)-based antigen detection, culture-based identification, F1 capsule antigen detection, PCR amplification, and virulence gene detection [115]. RBP proteins from *Y. pestis* phages φA1122 and L-413C were coupled to a fluorescent reporter protein to create a specific fluorescent probe for detecting bacterial cells under fluorescence microscopy. The initial observations confirmed that both tested proteins bound to *Y. pestis* cells after only 20 min of incubation. The authors then examined how culture temperature influenced the efficiency of binding. The results indicated that after 2 h incubation with bacteria in early logarithmic phase, RBP binding was observed at all tested temperatures (6, 20, 28, and 37 °C). However, the fluorescent signals for both RBPs were significantly weaker at 6 °C than at higher temperatures (20–37 °C for phage L-413C RBP and 28–37 °C for phage φA1122 RBP) [115]. The specificity of this test was confirmed using *Y. pestis* and other *Yersinia* species as a control, and the assay was performed under different growth and capsule-inducing conditions [115].

### 2.5. Pseudomonas aeruginosa Detection

*Pseudomonas aeruginosa* is a significant problem in healthcare systems. Infections caused by this bacterium are problematic in ICUs and are associated with high morbidity and mortality. This pathogen is especially dangerous for people with pneumonia, chronic obstructive pulmonary disease, or cystic fibrosis. The World Health Organization put this microorganism on the priority list of bacterial pathogens for which the development of new drugs is urgently needed [116]. This bacterium is responsible for 10–15% of nosocomial infections worldwide [117]. Additionally, infections caused by *Pseudomonas* are difficult to treat due to antibiotic resistance and the ability of this pathogen to acquire resistance to different antimicrobial agents [118]. *P. aeruginosa*-related bloodstream infection, which is considered to be one of its most serious complications, has a mortality of 18–61% [62]. Efforts to develop rapid diagnostic tests for pathogen recognition is an important element of the fight against *P. aeruginosa*-induced infections. One proposed method used a recombinant phage tail fiber protein (P069) from phage PA1. P069 was composed of wild-type TFP, a six-histidine (6-His) tag at the C-terminus, and three lysines at the N-terminus. To confirm the detection of bacteria by interaction of the obtained fluorescent protein, the authors performed TRITC (tetraethyl rhodamine isothiocyanate) labeling. The interaction of *P. aeruginosa* PA1 with the fluorescently labeled protein was observed under fluorescence microscopy. P069 was further functionalized with AffiAmino magnetic particles and incubated with *P. aeruginosa* to show the potential of magnetic separation as a tool for specific bacterial detection. The usefulness of P069 for efficiently detecting *P. aeruginosa* was confirmed in human urine, glucose, and rat serum samples. Together, these P069-based bioluminescent and fluorescent methods detected *P. aeruginosa* with lower limits of 6.7 × 10^2^ CFU/mL and 1.7 × 10^2^ CFU/mL, respectively [119].

### 2.6. Enterococcus *spp.* and Staphylococcus *spp.* Detection

*Staphylococcus aureus* is a major human pathogen that causes a wide range of diseases and is the leading cause of healthcare-associated infections [120]. *S. aureus* can colonize healthy individuals: approximately 30% of humans are asymptomatic nasal carriers of this bacterium [121], and *S. aureus* carriers have an increased risk of infection and are presumed to be an important source for the spread of *S. aureus* among individuals [122]. Epidemiological data indicate that the population incidence of bacteremia caused by *S. aureus* ranges from 10 to 30 per 100,000 persons per year [123]. This bacterium is known for its ability to become resistant to antibiotics, and this has become an epidemiological problem on a global scale. Diseases induced by methicillin-resistant *S. aureus* strains (MRSA) often occur in epidemic waves initiated by one or a few successful clones; these waves are problematic in both healthcare and community settings [122,124].

*Enterococcus* spp. lives harmlessly in the digestive tract, but if it spreads to other parts of the human body, it can cause serious health problems. Hospitals are the most common source of these infections. The use of more intensive and invasive medical therapies for humans has caused enterococcal infections to become more common. Increasing antibiotic resistance has also been noted among clinical isolates of enterococci. Many healthcare-associated strains have become resistant to vancomycin, penicillin, and aminoglycosides. *Enterococcus faecium* is more antibiotic-resistant than *Enterococcus faecalis*; more than half of the pathogenic isolates of the former exhibit resistance to different drugs [125]. Enterococcal infections are often responsible for urinary tract infections among hospitalized patients [126], along with intra-abdominal, pelvic, and soft tissue infections [127], bacteremia [128], and endocarditis [129]. Prompt diagnosis of enterococcal infection is essential for efforts to slow disease progression. One possible strategy is to use the lab-on-chip platform, which offers the benefits of low sample consumption and the possibility for fast and simple analysis [61]. The platform was combined with phage RBPs specific for *Enterococcus* spp. (gp18) and *Staphylococcus* spp. (gp109) for detection of nosocomial pathogens. The utilized proteins had two distinct domains: a C-terminal domain responsible for substrate recognition and receptor binding, and an N-terminal domain engineered to have a 6-His tag [130]. The N-terminal 6-His tag could form a stable complex with heavy metals, such as nickel, and Ni-magnetic beads were used to enable the oriented attachment of the phage RBP [131]. For analysis, bacterial cells were labeled with magnetic nanoparticles functionalized with specific phage proteins, which recognize phage RBP immobilized at the magnetoresistive chip surface. Finally, the magnetically labeled cells were detected by an array of spin-valve sensors on the biochip. This procedure was reported to take less than 2 h and detect both pathogens at concentrations in the range of 10 CFU/mL [132].

### 2.7. Salmonella *spp.* Detection

Each year, *Salmonella* infection causes 93.8 million cases of gastroenteritis and 155,000 deaths around the world [133]. It is recognized as the second most common zoonotic pathogen in Europe [134]. *Salmonella enterica* subsp. *enterica* serovar Enteriditis (*S. enteriditis*) and *Salmonella* Typhimurium are responsible for 60% and 30%, respectively, of all reported cases of human salmonellosis in the EU [135]. Members of the genus *Salmonella* belong to the family *Enterobacteriaceae* and are Gram-negative, facultative anaerobic bacilli. The genus is composed of two species, *S. enterica* (with six subspecies) and *Salmonella bongori*, and may be further subdivided into serotypes based on the presence of specific surface molecules, namely H-antigen (H-Ag, typically the major protein of the flagellar complex), flagellin, and O-antigen [136]. Members of *Salmonella* cause a well-characterized spectrum of diseases in humans, ranging from asymptomatic carriage to fatal typhoid fever. In the developed world, foodborne acute gastroenteritis and enterocolitis are the most common forms of *Salmonella* infection [66]. Under stress conditions, cells of *S.* Typhimurium tend to enter a metabolic starvation mode and may remain in a VBNC state. Bacteria in this state cannot be cultivated on conventional culture media but may still show high virulence. Detection of these pathogens has become a major challenge for food safety [137]. All conventional microbiological methods are highly selective and sensitive, but the use of culture-based biochemical and serological assays is time-consuming, laborious, and expensive [138]. The entire process can take at least 5 days to reach a diagnosis [139]. As a promising potential approach, researchers have proposed using biosensors to detect bacterial cells or toxins [67]. The combined use of a biosensor with bacteriophages or their products that can specifically bind *Salmonella* would conceivably be much easier and faster than the conventional methods.

Singh et al. (2010) reported new methods for the sensitive and selective detection of *S.* Typhimurium using genetically engineered TSP from phage P22. In the first such method, TSP mutated to have a cysteine at the N- or C-terminus (to abolish endorhamnosidase activity) were immobilized on a gold substrate. The authors incubated this system with *S.* Typhimurium in liquid medium for 20 min, and then detected bacterial cells with SEM or by fluorescence using SYTO staining. These proteins were more efficient in binding bacteria than whole P22 phage or the corresponding wild-type TSP, most likely due to the lack of enzymatic activity, which can cause cell lysis. Three *E. coli* strains tested to assess selectiveness showed negligible binding and the presence of non-host bacteria with host bacteria did not interfere with the probe activity, supporting the validity of this method. For real-time analytical detection using surface plasmon resonance (SPR), TSP were combined with gold substrate on SF-10 glass plates. Bacteria flowed at 100 µL/mL for 30 min. A concentration of *S.* Typhimurium as low as 10^3^ CFU/mL yielded a significant signal. The presence of an *E. coli* strain did not alter the binding activity for *Salmonella*, confirming the selectiveness of this assay. The mutated TSP further showed resistance to drying with N_2_, whereas whole-phage P22 lost its ability to bind the host bacteria after this process [138].

Hyeon et al. (2021) recently used full-length Det7 phage tail protein (Det7T) in a new SPR-based method for the rapid and selective detection of *S.* Typhimurium [140]. Det7T exhibited 50% sequence identity to the tail endorhamnosidase of *Podovirus* P22 [141], and both of these proteins bound octasaccharide fragments from *Salmonella* LPS. In addition to its strong infection ability, Det7T offered the potential for binding additional *Salmonella* serovars, exhibiting a combined susceptibility of approximately 60% across all *Salmonella* strains [142]. The developed biosensor system was composed of Det7T loaded to the gold-coated surfaces of a CM5 chip. Binding kinetics were observed for up to 5 × 10^7^ CFU/mL of *Salmonella*. This system exhibited no binding to non-host *E. coli* K12 cells. On SPR, the Det7T signal intensities were 10 times higher than those obtained with mutated P22 tailspike protein [138].

Hyeon et al. (2021) further examined the applicability of this biosensor by testing it against 10% apple juice spiked with *S.* Typhimurium cells. A notable signal was obtained across 5 × 10 to 5 × 10^7^ CFU/mL. The low detection sensitivity observed in this experiment most likely reflected that oligosaccharide fragments in juice could compete with sensor binding sites. The addition of a pretreatment step that removes additional saccharides might increase the accuracy of this method. Nonetheless, the reported results indicate that a biosensor with Det7T could be a useful tool for the rapid and selective monitoring of *S.* Typhimurium in environmental and food samples.

LTFs such as those from phage S16 have also been used for the rapid and specific immobilization and detection of *S.* Typhimurium. Denyes et al. (2017) used a gp-37 and gp-38 protein complex in combination with metal beads. After the conditions were optimized, the authors tested their system against various concentrations of *S.* Typhimurium DB7155 (from 10 to 10^5^ CFU/mL). The results showed that the level of recovery was 98%. Comparison of nine additional strains of *Salmonella* and several monophasic *S.* Typhimurium isolates revealed that the LTF-metal beads could recover 81–97% of the cells. In contrast, less than 5% recovery was observed for *E. coli* K12, *Citrobacter freundii*, *Cronobacter sakazakii* (which have similar Gram-negative structures) and *S.* Typhimurium DB7155 without LTF. The recovery ability for *S.* Typhimurium was not affected by the use of a mixture of different foodborne pathogens. The LTF–metal beads also proved useful in recovering *S.* Typhimurium DB7155 mixed with six food samples (milk, chocolate milk, RIF (reference infant formula), chicken, celery, alfalfa sprouts) at concentrations of 0, 1 to 10, 10, 100, and 1000 CFU per 25 mL or g. *Salmonella* CFU were detected in all samples containing at least 10 CFU/25 g or ml. In chicken, RIF, chocolate milk, and milk, contamination was detected at 1 to 10 CFU/25 g or ml. In terms of the USDA (United States Department of Agriculture) Microbiology Laboratory Guidebook and the FDA Bacteriological Analytical Manual, these results meet the required detection limit for *Salmonella* [139,143] while reducing the detection time from 72 h to 24 h in comparison with the current culture-based methods.

Denyes et al. (2017) further introduced an enzyme-linked LTF assay (ELLTA) that used LTF conjugated with horseradish peroxidase (HRP-LTF). This conjugate was coated onto the metal beads, which were incubated with *Salmonella* cells for 30 min. The unbound HRP-LTF was removed and a buffer containing 3,3’, 5,5’-tetramethylbenzidine (TMB) was added. This caused *Salmonella*-containing samples to turn visibly blue. To verify the sensitivity of this test, the authors tested bacterial dilutions from 10 to 10^8^ CFU/mL of *S.* Typhimurium DB7155 and used LTF-metal beads incubated with no cells and empty beads as controls. Bacteria-free controls exhibited a negligible amount of TMB (assessed by absorbance at 450 nm; A450). ELLTA was found to be sensitive, yielding significant results against as little as 10^2^ CFU/mL. The limit of detection for the reliable and practical detection of *Salmonella* was determined to be 0.49 A450, such that a sample with a value greater than 0.49 A450 was considered to be positive for *Salmonella*. The authors further tested the possibility of using ELLTA to rapidly count *Salmonella* cells. Indeed, measurement of A450 values for dilutions of 10 to 10^8^ CFU/mL *S.* Typhimurium DB7155 revealed a linear relationship of log10 CFU/mL to A450 readings between 10^5^ and 10^7^ CFU/mL, suggesting that this method could be appropriate for counting *Salmonella* cells within this concentration range [135].

Finally, the authors tested the specificity of ELLTA at a concentration of 10^5^ CFU/mL. Cross-genus *Salmonella* strains from food and clinical isolates, including five monophasic *S.* Typhimurium strains that are difficult to identify using routine tests and 10 non-*Salmonella* bacteria (Gram-positive and -negative), were checked. The results revealed that all of the *Salmonella* strains could be detected at values ranging from 0.62 to 3.02 A450. The 10 non-*Salmonella* strains had values below the level of detection. A detection efficiency score was determined by dividing the A450 value by the initial cell number, enabling the authors to rank the individual strains by the detection ability of HRP-LTF. All of the non-*Salmonella* strains had negative or near-zero detection efficiencies. The observed fluctuations in the ability of these strains to interact with HRP-LTF resulted from differences in the structure of cell surface [135].

In sum, this method could detect *S.* Typhimurium at concentrations from 10^2^ CFU/mL as well as 20 other *Salmonella* strains in just 2 h [135]. In comparison, conventional culture-based detection takes 5–7 days [143] and requires higher cell concentrations.

### 2.8. Shigella Detection

Humans represent the natural reservoir of *Shigella* spp., but increasing numbers of resistant strains are also found in livestock farming [144]. As *Shigella* is transmitted by the fecal–oral route, most cases of foodborne disease are associated with poor hygiene in food processing and preparation, especially in developing countries. Outbreaks commonly occur when food is exposed to a limited heat treatment or served/delivered raw to the consumer. Food products from which *Shigella* has been isolated include potato salad, ground beef, bean dip, raw oysters, fish, and raw vegetables [145].

The genus *Shigella* comprises four named species: *Shigella dysenteriae* (15 serotypes), *Shigella flexneri* (8 serotypes), *Shigella boydii* (18 serotypes), and *Shigella sonnei* (1 serotype). These four strains do not produce flagellins or capsular antigens and are subdivided into serotypes based on their O-antigens [146]. *S. flexneri* serotype 2a is the most prominent in developing countries [68]. *Shigella* is closely related to *E. coli* (EIEC) (enteroinvasive *E. coli*), complicating the design of a method to correctly detect the cause of an infection. Typical symptoms of *Shigella* infection include bloody diarrhea, abdominal pain, fever, and malaise [145]. Importantly, *S. flexneri* can cause an infection at only 10 CFU/mL, which is below the limit of detection for the current detection methods. The efficient detection of *Shigella* is therefore limited by the time needed to apply bacterial enrichment [147].

Various standardized methods have been developed to isolate and identify *Shigella* spp. from food samples. Some are included in the Bacteriological Analytical Manual (BAM) of the US FDA. Sf6 is a *Podovirus* phage for which *S. flexneri* serotype Y is a host. The tailspike protein from phage Sf6 (Sf6TSP) shows highly specific binding to a polysaccharide on the surface of this bacterium and has been used to develop methods for identifying *S. flexneri* [148]. Although Sf6TSP is an adhesin, it also exhibits enzymatic properties: it can act as an endorhamnosidase, cleaving α-(1→3) linkages between two rhamnoses. The authors used these properties to develop two methods involving SfTSP and reported that their engineered Sf6TSP could: (1) be applied in a rapid microtiter plate-based assay; and (2) be used as a sensitive fluorescent probe to enable detection.

Given the high specificity of Sf6TSP, the authors first tested whether it could be a suitable probe for *S. flexneri* in an ELISA-like tailspike adsorption assay (ELITA). For this purpose, they cloned an enzymatically inactivated Sf6TSP E366A/D399A carrying an N-terminal Strep-tag^®^II (StrepII-Sf6TSP). This was tested against four strains of *S. flexneri* and control strains of *E. coli* and *S.* Typhimurium. Bacteria were grown to stationary phase and adsorbed to the surface of a plate coated with StrepII-Sf6TSP. For detection of the modified protein, the authors used HRP-Strep-Tactin^®^. Positive identification was obtained for all four tested strains of *S. flexneri*, whereas no signal was obtained for *E. coli* or *S.* Typhimurium. As a reference control, the authors used P22TSP, which is a TSP known to bind *S. Typhimurium*. Tests performed using Sf6TSP showed results comparable to those obtained with P22TSP. The efficiency in the proposed *Shigella* detection method turned out to be higher or comparable to that used for the detection of *Salmonella*, depending on the *S. flexneri* strain [148,149]. To confirm that the O-antigen is responsible for the binding of the bacterium with the phage adhesin, the authors first incubated the bacteria with an enzymatically active Sf6TSP to remove the bacterial O-antigen. When ELISA was performed with Sf6TSP-treated bacterial cells, the obtained signals were reduced to 24–62% of those obtained with the non-pretreated cells, depending on the *S. flexneri* strain. To assess the serotype specificity of Sf6TSP, the authors added a Y polysaccharide preparation to the ELITA, setting up competition between the cell-surface O-antigen and free O-antigen polysaccharides in the solution. The Sf6TSP probe was quenched by the free antigens and could not bind to the bacterial surface. The results obtained showed that ELITA-based detection of *S. flexneri* was an exclusively O-antigen-specific process and the TSP did not bind to any other part of the target bacterium.

Kunstmann et al. (2018) also reported a fluorescent detection method for *S. flexneri*. For this purpose, the authors designed a specific Sf6TSP probe in which labeling with the environment-sensitive dye, N-methyl-N-[2-[methyl(7-nitro-2,1,3-benzoxadiazol-4-yl)amino]ethyl] (NBD), was used to generate a signal only upon O-antigen binding. The Sf6TSP probe was coupled with NBD via cysteine residues. When the labeled protein complex bound to the bacterial cell, fluorescence emission was increased as expected [150]. Kunstmann et al. (2018) found that the binding rate of the modified protein did not differ from that of the unlabeled protein, suggesting that this method could be applicable for detecting the *S. flexneri* O-antigen.

### 2.9. Bacillus anthracis Detection

*Bacillus anthracis* is an etiological factor of anthrax. It is a worldwide zoonosis caused by a Gram-positive, spore-forming bacterium. Infections in humans are caused by contact with animals, particularly grazing herbivores, and with animal products such as wool. Anthrax takes one of three forms [151,152], and the most common infection type is cutaneous anthrax; this form occurs in about 90% of cases. The other two forms are gastrointestinal and pulmonary anthrax [151]. *B. anthracis* is a dangerous pathogen and is considered to be a biological weapon. For this reason, a rapid detection method should be developed. The gold standard for *B. anthracis* testing is still the PCR method, which targets genetic markers (dhp61, PL3, plcR). For rapid pathogen detection, e.g., in the field, lateral flow assays (LFAs) are used. Unfortunately, this method does not seem to be highly sensitive or specific [69]. 

Braun et al. (2021) proposed a *B. anthracis* detection method based on phages RBPs. The authors developed two enzyme-linked phage RBP assays called ELPRA. Both tests were based on BA4079 protein (named RBP_λ03_) encoded by lambdoid prophage 03 located on the chromosome of *B. anthracis*. The N-terminally truncated version of the protein (RBP_λ03Δ1-120_) was also proposed, which was shown to bind specifically to *B. anthracis* cells in different growth phases. The first presented method was the Colony Lift and Blot ELPRA—in this method, the authors grew overnight bacterial cultures on agar plates; next, the colonies were blotted onto hydrophobic nitrocellulose membranes. To detect bacterial colonies, NanoLuc-RBP_λ03Δ1-120_, RBP conjugated with commercial luciferase, was used (NanoLuc from Promega). To confirm the binding of the modified protein on the membrane of the bacterial cells, Nano-Glo^®^ Luciferase Assay Substrate from Promega was used and luminescence was recorded on a ChemiDoc MP imaging system. Researchers used this assay to detect colonies of *B. anthracis* in a mixed culture plate with *B. cereus* and were able to distinguish *B. cereus* from *B. anthracis* as only the colonies of *B. anthracis* exhibited luminescence due to specific binding of NanoLuc-RBP_λ03Δ1-120_. In addition, a spiked soil sample was tested to identify *B. anthracis* in a heterogeneous environmental culture and the results were promising: only *B. anthracis* colonies were detected, with only one strain of *Bacillus* resulting in a false positive. As stated by the authors, if the process is started at the colony lift step, the assay takes about 1.5–2 h [69].

The second method was called the Rapid Dichotomous Colorimetric ELPRA. For this assay, the authors prepared two protocols: a two-step, indirect ELPRA and a one-step direct ELPRA. In the two-step ELPRA, RBPs fused with mCherry and TwinStrepTag (TST) were used. First, the colonies from agar plate were lifted with a loop and resuspended in the PBS in test tube, and following several washing steps, Strep-Tactin® horseradish peroxidase conjugate was added to the samples. For detection, SeramunBlau® slow peroxidase substrate (containing 3,3′,5,5′-tetramethylbenzidin) was used and the development of a blue signal was monitored [69]. This was relatively fast since the assay, which includes the wash steps, could be completed under 30 min. In the one-step version of this method, the HRP moiety was directly conjugated to the RBP_λ03Δ1-120_ reporter protein. When HRP-RBP_λ03Δ1-120_ was tested on *B. anthracis* and *B. cereus* colony material, only *B. anthracis* yielded blue signals.

The two presented procedures for *B. anthracis* detection, the colony lift and blot ELPRA, have potential to be a novel tool for rapid pathogen identification. The ELPRA implementation linking the RBP has the potential to be a rapid colorimetric method for specific bacteria detection, supporting PCR-based testing. The specificity of this procedure is high with an overall specificity of >95%. Together with the possibility of obtaining the result in a few minutes, this means that these protocols can be used in rapid diagnostic testing.

### 2.10. Klebsiella pneumoniae Detection

*Klebsiella pneumoniae* is a well-known multidrug-resistant pathogen and has been declared a global concern of top priority. According to the European Centre for Disease Prevention and Control (ECDC) report from 2019, *Klebsiella* spp. Strains were among the three strains that most often caused pneumonia in patients undergoing intensive care [153,154]. In addition, a report from the World Health Organization (WHO) indicates that approx. 50% of detected *K. pneumoniae* strains are multidrug-resistant [155,156]. Time-consuming and mainly culture-based methods are primarily used to detect infection caused by this pathogen. It is known that infection with this pathogen leads to rapidly progressing pneumonia and sepsis. Therefore, promising *K. pneumoniae* infection detection tools are phages and phage-derived proteins.

Nogueira et al., 2021 reported new isolated *Klebsiella* phage KpnM6E1. The authors also identified and characterized a novel RBP gp86. First, gp86 fused with mCherry was produced. Next, specificity and selectivity of protein binding to the cells were studied by epifluorescence microscopy. The presented results suggested that gp86 RBP was specific for *K. pneumoniae*, and red fluorescence was observed only for this strain. To confirm the binding of the protein, RBP-based fluorescence spectroscopy was used. This method was recently developed by the group for multiplex detection of *Staphylococcus* and *Enterococcus* [129]. The obtained results supported the data collected from the microscopy assays. For other species tested, such as *Staphylococcus* spp., *A. baumannii*, *Enterococcus* spp., or *P. aeruginosa*, no fluorescence was detected. The sensitivity of this test was high, and RBP was able to recognize 80% of *K. pneumoniae* strains [70].

As the authors suggested, the novel RBP presented in Nogueira et al. 2021 provided a sensitive and specific biorecognition molecule for *K. pneumoniae* detection. As presented in this paper, RBP was fused with a fluorescence tag which allowed for the use of the RBP in fluorescence microscopy, ELISA, or spectrofluorometry. Additionally, gp86 can be used in other biosensor assays, for example, SPR, surface-enhanced Raman scattering (SERS), label-free long period gratings (LPGs), or microwave sensors [70].

## 3. Conclusions

Bacteriophages combine several properties that are desirable for the purpose of detecting bacterial pathogens. In this review, the authors focused on capture-dependent methods in which phage tail proteins are used to detect pathogenic bacteria. The reviewed methods include numerous examples in which phage RBPs are used to rapidly and specifically detect hosts. The described methods include colorimetric, fluorescent, SPR, and microscopy-based detection strategies. Compared to the traditional culture-based methodologies, capture-dependent methodologies are accurate, reliable, simple, relatively inexpensive, fast, and require a fairly low skill level. These properties are all desirable for diagnostics, suggesting that phage tail protein-based capture methods could potentially improve the treatment and control of pathogenic bacteria, thereby decreasing their negative impact worldwide.

## Figures and Tables

**Table 1:** Overview of methods based on RBP or TFP from different bacteriophages.

Target Species	Capture Method	Detection (Visualization) Method	Limit of Detection	Reference
*Acinetobacter baumannii*	Sandwich fluorescence assay	Fluorescence (FITC-labeled probes)	6.2 × 10^2^ CFU/mL	[58]
	Magnetic beads coated with TFP	Bioluminescence (ATP release with luciferin/luciferase detection)		
	Magnetic nanoparticles coated with TFP	MALDI-TOF MS	∼2.34 × 10^5^ and ∼4.48 × 10^4^ CFU/mL, depending on the strain	[59]
*Acinetobacter baumannii Pse* + other *Pse* bacteria (Pse–Ppseudoaminic acid)	Incubation in solution	Fluorescently labeled probe	-	[60]
	TFP-coated microplate wells	FITC labeling of bacteria		
*Enterococcus faecalis*, *Enterococcus faecium*	Magnetic nanoparticles coated with His-tagged TFP	Array of spin-valve sensors on the biochip	10 CFU/mL	[61]
*Staphylococcus aureus*				
*Pseudomonas aeruginosa*	Magnetic particles	Magnetic separation	6.7 × 10^2^ CFU/mL and 1.7 × 10^2^ CFU/mL	[62]
	Fluorescent labeling by TRITC	Fluorescent microscopy		
*Yersinia pestis*	Fluorescent probe (RBP proteins from *Y. pestis* phages φA1122 and L-413C)	Fluorescent microscopy	-	[63]
*Campylobacter jejuni*, *Campylobacter coli*	Microresonator functionalized with the GST-Gp48 tailspike	Biosensors	-	[64]
	RBP and GFP-coupled RBP (Gp047)	Agglutination assay on glass slide and fluorescent microscopy	-	[65]
*Salmonella* spp.	TSP-coated gold/incubation in solution	SEM or SPR	10^3^ CFU/mL in case of SPR	[66]
	Det7T loaded to the gold-coated surfaces of a CM5 chip	SPR	5 × 10^7^ CFU/mL	[67]
	Metal beads conjugated with HRP-LTF; incubation in solution	enzyme-linked LTF assay (ELLTA)	10^2^ CFU/mL	
*Shigella* spp.	Sf6TSP cloned with Strep-Tag coated microplate wells	ELISA-like tailspike adsorption assay (ELITA)	10^3^ CFU/mL	[68]
*Bacillus anthracis*	NanoLuc-RBP_λ03Δ1-120_, RBP conjugated with commercial luciferase	Enzyme-linked phage receptor binding protein assay (ELPRA)-luminescence	-	[69]
	HRP moiety directly conjugated to the RBP_λ03Δ1-120_	ELPRA-colorimetric assay		
*Klebsiella pneumoniae*	Gp86 RBP fused with mCherry	Fluorescence microscopy and RBP-based fluorescent spectroscopy	-	[70]

Legend: GFP—green fluorescent protein; CFU—colony forming unit; TFP—tail fiber protein; SEM—scanning electron microscopy; SPR—surface plasmon resonance; ELPRA-enzyme-linked phage receptor binding protein assays; FITC—fluorescein isothiocyanate; MALDI-TOF MS—Matrix-Assisted Laser Desorption Ionization–Time of Flight Mass Spectrometry; RBP—receptor binding protein; TSP—tailspike proteins; HRP—horseradish peroxidase.

## Data Availability

Not applicable.
